# Chlorpromazine for schizophrenia: a Cochrane systematic review of 50 years of randomised controlled trials

**DOI:** 10.1186/1741-7015-3-15

**Published:** 2005-10-17

**Authors:** Clive Elliott Adams, John Rathbone, Ben Thornley, Mike Clarke, Jo Borrill, Kristian Wahlbeck, A George Awad

**Affiliations:** 1 Cochrane Schizophrenia Group, Academic Department of Psychiatry and Behavioural Sciences, University of Leeds, 15 Hyde Terrace, Leeds, LS2 9LT, UK; 2 UK Cochrane Centre, Summertown Pavilion, Middle Way, Summertown, Oxford, OX2 7LG, UK; 3 Safer Custody Group, HM Prison Service, Abell House, John Islip Street, London, SW1P 4LH, UK; 4 STAKES/Vasa Central Hospital, Department of Psychiatry, FIN-65130 Vaasa, Finland; 5 University of Toronto, Humber River Regional Hospital, Keele Street Site, 2175 Keele Street, Toronto, Ontario, M6M 3Z4, Canada

## Abstract

**Background:**

Chlorpromazine (CPZ) remains one of the most common drugs used for people with schizophrenia worldwide, and a benchmark against which other treatments can be evaluated. Quantitative reviews are rare; this one evaluates the effects of chlorpromazine in the treatment of schizophrenia in comparison with placebo.

**Methods:**

We sought all relevant randomised controlled trials (RCT) comparing chlorpromazine to placebo by electronic and reference searching, and by contacting trial authors and the pharmaceutical industry. Data were extracted from selected trials and, where possible, synthesised and random effects relative risk (RR), the number needed to treat (NNT) and their 95% confidence intervals (CI) calculated.

**Results:**

Fifty RCTs from 1955–2000 were included with 5276 people randomised to CPZ or placebo. They constitute 2008 person-years spent in trials. Meta-analysis of these trials showed that chlorpromazine promotes a global improvement (n = 1121, 13 RCTs, RR 0.76 CI 0.7 to 0.9, NNT 7 CI 5 to 10), although a considerable placebo response is also seen. People allocated to chlorpromazine tended not to leave trials early in both the short (n = 945, 16 RCTs, RR 0.74 CI 0.5 to 1.1) and medium term (n = 1861, 25 RCTs, RR 0.79 CI 0.6 to 1.1). There were, however, many adverse effects. Chlorpromazine is sedating (n = 1242, 18 RCTs, RR 2.3 CI 1.7 to 3.1, NNH 6 CI 5 to 8), increases a person's chances of experiencing acute movement disorders, Parkinsonism and causes low blood pressure with dizziness and dry mouth.

**Conclusion:**

It is understandable why the World Health Organization (WHO) have endorsed and included chlorpromazine in their list of essential drugs for use in schizophrenia. Low- and middle-income countries may have more complete evidence upon which to base their practice compared with richer nations using recent innovations.

## Background

Chlorpromazine is in the World Health Organization (WHO) list of essential drugs [1]. It is estimated that 24 million people currently suffer from schizophrenia [2], the majority of whom live in low or middle-income countries. Until recently, it would have been common practice for anyone with schizophrenia to have been treated with chlorpromazine at some point [3,4]. Despite well-documented adverse effects, and the advent of a new generation of antipsychotic drugs, chlorpromazine remains one of the most commonly used and inexpensive treatments for people with schizophrenia [5]. In Africa, chlorpromazine was widely used [6], although we have failed to identify any more recent surveys. In India chlorpromazine is commonly prescribed, and in South East Asia the older generation of antipsychotics are used to treat the majority of people with schizophrenia [7]. In 2003, in the UK, chlorpromazine was the most frequently prescribed of the first generation 'typical' antipsychotic drugs, where, at that time, the 'typical' group of antipsychotics accounted for 44% of all anti-psychotic prescriptions [8].

As well as its almost universal use in clinical practice, chlorpromazine is a benchmark by which other treatments are evaluated [9]. There are many qualitative reviews of chlorpromazine but few attempts have been made to quantify data from randomised controlled trials (RCTs) [10,9,11]. An up-to-date quantitative review of the effects of this old, highly prevalent treatment is long overdue. 50 years after its formulation, the evidence should be more complete than when the drug was under patent.

## Methods

### Inclusion criteria

The inclusion criteria were defined and disseminated for peer review within a Cochrane protocol first published 1998 [12]. Articles were included if they reported RCTs where the participants had schizophrenia or non-affective serious/chronic mental illness, and where the interventions included chlorpromazine (any dose or mode of administration) versus placebo or no treatment.

### Identification of relevant trials

We identified relevant randomised trials by searching the Cochrane Schizophrenia Group's register of trials (June 2002), with a phrase designed to identify the many ways of naming chlorpromazine [see Additional file 1]. Citations in all identified articles were inspected for further trials. Rhône-Poulenc Rorer (the original distributors of chlorpromazine) was contacted to request access to archive material, and Dr RA Pargiter (Hobart, Tasmania) donated a large series of May and Baker chlorpromazine reports from 1955 to 1973.

### Data extraction and study appraisal

All electronic records identified were independently inspected by BT, CA and JR. The reliability of selection processes and data extraction was checked using a 10% random sample. Full reports of studies of agreed relevance were obtained, quality rated [13], and data relating to methods, participants, interventions and outcomes, extracted. Any disagreement was discussed and decisions documented. If there were outstanding issues, the authors of the studies were contacted where possible to help resolve problems.

### Statistical methods

Dichotomous and continuous data were not used if over half of those randomised did not contribute to the outcome due to early attrition from the study or non-compliance. Dichotomous data were combined using a random effects Relative Risk (RR) [14]. Numbers needed to treat/harm (NNT/H) [10] were also calculated, and χ^2 ^tests for heterogeneity were performed. Where <50% of people were lost to follow-up at the end of a trial, 'worst case' intention-to-treat analyses were undertaken by assuming that those who had left a trial early had had a poor outcome. The sensitivity of the final results to this assumption was tested. Continuous data were excluded if derived from scales of unknown validity and if totals or measures of variance were not reported. Summation was not attempted where continuous data were too skewed [15]. All estimates of effect are presented with their 95% confidence intervals (CI).

## Results

Electronic searches identified over 1000 records, most of which were ineligible. Full copies of 351 citations were obtained for detailed scrutiny, including a further 50 papers identified from citations. Of these, 302 papers were excluded and 99 reports of the 50 RCTs included (Table 1). Studies were mainly excluded due to lack of random allocation (68%). However, 43 randomised trials (30%) reported irrelevant outcomes, such as serum levels of chlorpromazine breakdown products, or presented data in such a way as to make the outcomes unintelligible or impossible to use.

**Table 1:** Included studies.

		**METHODS**				**PARTICIPANTS**					**INTERVENTIONS**			**OUTCOMES**						
**INCLUDED STUDIES (date of publication)**		**Randomised**	**Double-blind**	**Three+ arm study**	**Duration (weeks)**	**Only Schizophrenia**	**History**	**Total number of participants**	**Age (years)**	**Sex**	**CPZ dose (mg/day)**	**Number allocated CPZ**	**Number allocated placebo**	**Leaving the study early**	**Global improvement**	**Mental State**	**Side-effects**	**Global clinical state**	**Behaviour**	**Relapse**

**1955**	**Hall**	●	●		9	●	C	175	20–59	M+F	**750 max**	**87**	**88**	●	●		●		●	
	**Vaughan**	●	●		U/K		C	48	M = 43	F	**75–450**	**24**	**24**		●					
**1956**	**Shepherd**	●	U/K	●	6	●	C	24	27–52	F	**300**	**8**	**8**	●	●					
**1958**	**Abrams**	●			4	●	C	40	20–55	F	**200–600**	**20**	**20**	●						
	**Grygier**	●	U/K		24	●	C	30	m = 50	F	**150**	**15**	**15**		●					
	**Hine**	●	●		20	●	C	22	30–50	F	**750 max**	**11**	**11**	●	●		●			
	**Simon**	●		●	4		U/K	80	m = 31	U/K	**200–1200**	**20**	**0**	●						
**1959**	**Baker**	●	●	●	5		C	25	33–79	F	**150–300**	**7**	**7**	●	●	●	●			
	**Flemming**	●	●	●	26	●	C	63	m = 58	F	**75–300**	**21**	**21**	●			●		●	
	**Walsh**	●	U/K	●	8	●	C	66	27–50	F	**75–300**	**22**	**22**		●		●			
**1960**	**Englhardt**	●	●	●	78	●	U/K	173	18–40	U/K	**50–800**	**62**	**56**	●						●
	**Hamilton**	●	●	●	8	●	C	54	m = 38	M	**300**	**18**	**18**	●			●			
	**Payne**	●	●	●	6	●	C	21	23–73	M	**25–100**	**7**	**7**				●			
	**Somerville**	●	●	●	6		C+A	60	24–58	F	**200–800**	**15**	**30**	●	●		●		●	
**1961**	**Clark**	●	●	●	24	●	C	60	26–52	F	**200–800**	**20**	**20**		●		●			
	**Lorr**	●	●	●	12		A	308	<50	M	**50–100**	**63**	**61**	●						
	**Kurland**	●	●	●	6		A	277	18–61	M+F	**300**	**33**	**72**	●	●		●			
	**Schiele**	●	●	●	16	●	C	80	m = 80	M	**200–1000**	**20**	**20**	●		●	●	●		
	**Smith**	●	●	●	14	●	C	30	m = 42	M+F	**150–600**	**13**	**15**						●	
**1963**	**Bishop**	●	●	●	10	●	C	30	U/N	M+F	**800**	**10**	**10**	●	●					
	**Fink**	●	U/K	●	6		S	311	m = 31	M+F	**1200**	**51**	**44**	●						
**1964**	**NIMH**	●	●	●	6	●	A	463	16–45	M+F	**200–1600**	**112**	**125**	●						
**1966**	**Reardon**	●	●	●	4	●	A	34	U/K	M+F	**300–600**	**11**	**12**	●	●					
	**Saretsky**	●	●		12	●	A	40	<55	M	**400**	**20**	**20**	●						
**1967**	**Clark**	●	●		10	●	C	72	25–55	F	**678 m**	**51**	**21**	●						
	**Letemendia**	●	●		39		C	28	<65	M	**300**	**14**	**14**	●						
**1968**	**Clark a**	●	●	●	14	●	C	72	20–60	F	**1000 max**	**18**	**36**	●	●		●			
	**Clark b**	●	●	●	16	●	C	69	20–60	F	**1000 max**	**23**	**23**	●			●			
	**Cohen**	●	●	●	60	●	C	126	18–42	M+F	**180**	**42**	**42**	●						
	**Prien**	●	●	●	24	●	C	838	19–55	M+F	**2000**	**208**	**212**		●		●	●	●	●
											**300**	**208**								
**1969**	**Tetreault**	●	●	●	12	●	C	45	m = 50	F	**300–600**	**15**	**15**	●		●	●		●	
**1970**	**Clark a**	●	●	●	12	●	C	44	22–55	M+F	**200–1000**	**15**	**14**	●	●		●	●	●	
	**Clark b**	●		●	24	●	C	71	21–60	F	**150–600**	**54**	**18**	●			●		●	
**1971**	**Clark**	●	●	●	4	●	C	86	21–45	M+F	**200–1000**	**23**	**21**	●	●		●			
**1972**	**Clark**	●	●	●	12	●	C	55	21–60	M+F	**1000**	**19**	**18**	●	●		●	●		
	**Serafetinedes**	●	●	●	12	●	C	57	21–61	M+F	**1000 max**	**14**	**13**	●	●		●			
**1973**	**Hogarty**	●	●		156	●	S	374	18–53	M+F	**270 m**	**192**	**182**	●						●
	**Klein**	●	●		6	●		88	17–61	M+F	**300–1200**	**46**	**42**	●						
**1974**	**Reschke**	●	●	●	0.1	●	A	50	19–57	M+F	**25 im**	**10**	**11**	●	●		●			
**1975**	**Ban**	●	●	●	12	●	C+A	30	17–46	M+F	**200–800**	**10**	**10**	●	●					
	**Hamill**	●			0.7	●	A	44	18–55	M+F	**306–475**	**22**	**22**	●						
**1977**	**Clark**	●	●	●	12	●	C	27	23–61	M+F	**1000**	**9**	**9**	●	●		●			
	**Spohn**	●	●		6+	●	U/K	40	18–55	M+F	**200 min**	**20**	**20**							●
**1978**	**Rappaport**	●			U/K	●	A	127	16–40	M	**300–900**	**53**	**74**	●						●
**1981**	**Peet**	●	●	●	12	●	U/K	53	m = 51	M+F	**400 max**	**16**	**18**	●			●			●
**1982**	**Nishikawa**	●	●	●	156	●	S	55	m = 33	M+F	**75**	**10**	**10**							●
**1986**	**Zuoze**	●		●	4	●	C	60	m = 36	U/K	**450 m**	**20**	**20**					●		
**1990**	**Chouinard**	●	●	●	4	●	A	62	19–62	M+F	**300–1200**	**21**	**21**	●	●		●		●	
**1991**	**Borison**	●	●	●	4	●	A	30	22–58	M	**400–1600**	**9**	**10**			●		●		
**2000**	**Cooper**	●	●	●	8	●	C	159	18–42	M+F	**600**	**53**	**53**	●	●	●				

Key: ● = Yes; U/K = Unknown; C = Chronic; A = Acute; M = Male; F = Female; m = Mean; im = Intramuscular injection.

### Study quality

All 50 included studies reported the use of random allocation; only 4 were explicit about the process used. Two used the toss of a coin [16,17], and 2 used random number tables [18,19]. Citations to all included and excluded studies are available in the full Cochrane Review [12], otherwise the names and dates cited in this text relate to Table 1. A further 2 trials [20,21] described some form of allocation concealment (sealed envelopes in both cases). The other 44 studies gave little assurance that bias was minimised during the allocation procedure and this may mean that this review overestimates the effect of chlorpromazine [22]. Twenty-eight (56%) of the trials adequately described their attempts to be double-blind, with two [20] and [23] reporting how successful these attempts were. Two studies [18] and [24] gave no indication that blinding had been attempted. Other trials indicated that an attempt at blinding had been made, but they gave no description of how this had been done. The description of participants who left studies early was poor; 12 of the 50 included studies providing no details of treatment withdrawals. Presentation of data was also poor. Trials frequently presented both dichotomous and continuous data in graphs, or reported inexact statistical measures of probability, for example p > 0.05. This often made it impossible to extract raw data for synthesis. Continuous scale data were frequently collected in the trials, but were often poorly reported; 30/50 trials did not report standard deviations and 9/42 did not present any data from the scales they had used.

### Study designs

The studies were mostly either 6 or 12 weeks long, but the range was large (24 h to 3 years). The great majority of participants in nearly all of the trials were diagnosed as suffering from schizophrenia. These studies reported on >5276 people, 3318 of whom were allocated to chlorpromazine-placebo comparison. Eleven of the 50 trials described the diagnostic criteria used, or the symptoms required for people to be included. Otherwise entry to most of the included studies was based on a pragmatic diagnosis of schizophrenia. The trials ranged in size from 21 [25] to 838 participants [26]. Most people were hospitalised at the time of the study. The lowest dose of chlorpromazine tested was 25 mg/day [27] and the highest 2000 mg/day [26]. One trial [28] included both a placebo and a no-drug group, which we combined. Another study included both a placebo group and a "routine conventional hospital treatment" group [26]. Data from the latter were not used in this review, as people in this group will probably have been given antipsychotic drugs.

### Outcomes

Table 2 presents the main results of this review. These intention-to-treat data are derived by synthesising homogeneous trial findings. The results remain essentially unchanged when we only used data from participants who completed the studies. The data show no clear pattern indicative of publication bias when sorted by study size and effect [29].

**Table 2:** Results relating to clinical change and study attrition.

	**Months**	**Number of trials**	**Chlorpromazine**	**Placebo**	**RR (95% CI)**	**Test for heterogeneity**
			**events/total participants**			

Relapse	6–24	3	108/202	159/192	0.65 (0.5–9.0)	Chi^2 ^7.83, df 2, p = 0.02 I^2 ^= 74.5%*
No global improvement	2–6	13	470/654	406/467	0.76 (0.7–0.9)	Chi^2 ^25.4, df 12, p = 0.01 I^2 ^= 52.8%
Leaving the study early	6–24	2	38/254	33/238	1.09 (0.7–1.6)	Chi^2 ^0.47, df 1, p = 0.49 I^2 ^= 0%

* no clear cause of heterogeneity found on close re-inspection of trials

**Figure 1:**
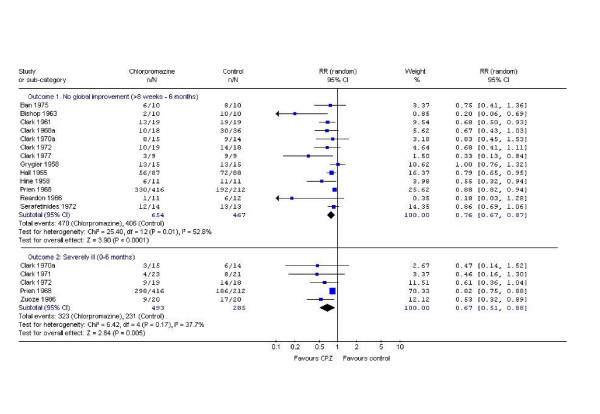
Chlorpromazine versus placebo – global outcomes.

Data on global improvement (a dichotomised impression of *change*), in the period up to 6 months favours chlorpromazine (n = 1121, 13 RCTs, RR No global improvement 0.76 CI 0.7 to 0.9) but is moderately heterogeneous (I^2 ^= 52.8%). Global severity of illness at study end (a dichotomised impression of clinical *state*) also favours chlorpromazine (n = 778, 5 RCTs, RR severely ill 0.67 CI 0.5 to 0.8, NNT 4 CI 3 to 10; Figure 1). Very few studies present usable data directly relating to end point mental state. The continuous data that are available (Brief Psychiatric Rating Scale [30] are equivocal (n = 49, 2 RCTs, RR -4.82 CI -8.5 to 1.2). Most information on behaviour relates to a dichotomous outcome of 'behaviour deteriorated/disturbed/uncooperative' (n = 1127, 10 RCTs, RR 0.53 CI 0.3 to 0.9) but these data are heterogeneous (χ^2 ^73, df 9, p < 0.00001).

Chlorpromazine has many adverse effects (Table 3). It is a sedative (n = 1242, 18 RCTs, RR 2.3 CI 1.7 to 3.1, NNH 6 CI 5 to 8) that may cause weight gain (n = 165, 5 RCTs, RR 4.44 CI 2.1 to 9.3, NNH 3 CI 2 to 5). Extrapyramidal symptoms are common and include acute dystonias (n = 780, 4 RCTs, RR 3.1 CI 1.3 to 7.7, NNH 24 CI 15–57) and Parkinsonism (n = 1265, 12 RCTs, RR 2.6 CI 1.2 to 5.4, NNH 10 CI 8 to 16). Data on chronic movement disorders such as tardive dyskinesia, however, are not available from this review as this requires longer follow-up than was attempted for nearly all the trials. Occurrence of akathisia is similar in the chlorpromazine and placebo groups. For every 7 people given chlorpromazine, one will experience some form of photosensitive reaction (n = 799, 6 RCTs, RR 5.19 CI 3 to 10, NNH 7 CI 6 to 10); hypotension and dizziness are common (n = 1232, 15 RCTs, RR 1.9 CI 1.4 to 27, NNH 12 CI 8 to 22); and dry mouth is considerably increased (n = 756, 5 RCTs, RR 4.00 CI 1.6 to 10, NNH 18 CI 13 to 37). Eye opacities, as identified by slit-lamp examination within one large trial using high dose chlorpromazine 2 gms/day [26], were increased within the drug group (n = 657, RR 3.09 CI 1.9 to 5.1, NNH 7 CI 5 to 10). There were no significant differences between people given placebo and those allocated chlorpromazine in the frequency of complaints of constipation, urinary retention and blurred vision.

**Table 3:** Adverse effects.

	**No. of trials**	**Chlorpromazine**	**Placebo**	**RR (95% CI) Random**
			
		**events/total participants**		
**General symptoms**				
Sedation	18	224/725	68/517	2.30 (1.7–3.1)
Weight gain > 10 lb; 4.5 Kg	5	31/75	7/90	4.44 (2.1–9.3)
**Extrapyramidal symptoms**				
Acute dystonia	4	28/472	5/306	3.10 (1.3–7.7)
Parkinsonism	12	123/723	40/542	2.60 (1.2–5.4)
Fits	3	19/450	4/245	2.41 (0.4–16.4)
Akathisia	8	53/602	40/400	0.95 (0.5–1.9)
**Allergic-type symptoms**				
Agranulocytosis/leucopenia	7	10/207	2/187	2.02 (0.7–5.6)
Rashes/itching	11	42/658	21/475	1.43 (0.9–2.4)
Jaundice	3	8/116	1/115	4.04 (0.9–17.9)
Photosensitivity	6	81/496	9/303	5.19 (2.7–9.8)
Eye opacity	2	97/431	16/226	3.09 (1.9–5.1)
**Anti-cholinergic/nor-adrenergic symptoms**				
Hypotension + dizziness	15	113/708	38/524	1.90 (1.4–2.7)
Constipation	9	40/590	16/365	1.68 (0.9–2.9)
Urinary retention	3	11/459	5/253	1.49 (0.5–4.3)
Dry mouth	5	32/473	4/283	4.00 (1.6–9.8)
Blurred vision	6	10/529	9/381	1.10 (0.5–2.9)

There were no reports of deaths occurring during any of the studies. Any data relating to violent incidents, hospital discharge or admissions, presence of delusions or hallucinations were either absent or impossible to use. Not one of the studies, even in recent years, reported levels of satisfaction and quality of life, nor could we identify any direct economic evaluation of chlorpromazine.

## Discussion

These 50 studies amounted to a total of >2000 person-years of exposure to chlorpromazine or placebo. For people with this serious mental illness, and certainly in situations were resources are limited, chlorpromazine remains a first line treatment. The medium term data on improvement suggest that about 7 people have to be treated for one to have what the trialists would describe as 'global improvement' (n = 1121, 13 RCTs, RR 0.76 CI 0.7 to 0.9, NNT 7 CI 5 to 10). This outcome relates to a simple dichotomised impression of a person's mental state, behaviour and functioning. Given the limited quality of reporting and the fact that we may be only able to pool data from a subset of the included trials, even this finding may be an over estimate of the positive and an underestimate of the negative effects of giving chlorpromazine.

The increased likelihood that people given chlorpromazine continued in their trial may be heartening. It could indicate a genuine decrease in the distressing symptoms of schizophrenia that led to an increased compliance with medication, despite common and unpleasant adverse effects such as sedation and hypotension. Doctors and nurses may, at times of acute disturbance, welcome this sedative effect but people with schizophrenia may not.

Despite limitations, this review provides quantitative evidence to confirm many of the impressions held by clinicians and recipients of care about the effects of chlorpromazine. Chlorpromazine is a sedating drug, prone to cause movement problems. Reliable evidence about its short-term effects is surprisingly weak, but information from studies that are >6 months does suggest that chlorpromazine facilitates a global improvement and may decrease the likelihood of behaving in a disturbed manner, at least within the confines of hospital.

## Conclusion

Chlorpromazine represents a low-cost choice for clinicians world-wide and merits its position as a benchmark treatment for psychotic symptoms. Until large, high quality, clinically relevant trials show equally inexpensive treatments to be both more effective and safe, chlorpromazine is likely to continue to be one of the most widely used treatments for the millions of people who suffer with schizophrenia.

Although the NNTs may seem high, and the NNHs low, these estimates are likely to be more realistic than those for new drugs, for which all evidence has not been made available. As time passes, studies not seen in the early years of marketing tend to become apparent. These studies may be systematically different from those initially used to sell the drug. As a result, clinical practice of low and middle-income countries, often having to use older generations of drug, may, nevertheless, have more chance of being based on all evidence than that of high income nations. The latter are prone to purchase new expensive innovations the evidence for which is treated with sensitivity by researchers, marketers, and licensing agencies mindful of pecuniary influences.

## Competing interests

The author(s) declare that they have no competing interests.

## Authors' contributions

BT participated in the protocol development, trial searching, data extraction, analysis, data interpretation and writing the manuscript.

JR participated in the trial searching, data extraction, analysis, data interpretation, writing the manuscript and maintaining the review.

CEA participated in the protocol development, trial searching, data extraction, analysis, data interpretation, writing the final manuscript and maintaining the review.

GA participated in the protocol development and data interpretation.

MC participated in the calculation and understanding of the results and production of the final manuscript.

JB participated in the calculation and understanding of results, and production of the final manuscript.

KW participated in the protocol development, calculation and understanding of the results and writing the final manuscript.

## Pre-publication history

The pre-publication history for this paper can be accessed here:



## Supplementary Material

Additional File 1 **Search strategy for identification of studies**. The Schizophrenia Group's register is based on regular searches of BIOSIS Inside; CENTRAL; CINAHL; EMBASE; MEDLINE and PsycINFO; the hand searching of relevant journals and conference proceedings, and searches of several key grey literature sources. A full description is given in the Group's module on the Cochrane Library. Click here for file
