# Supplementary material for: Chlorpromazine for schizophrenia: a Cochrane systematic review of 50 years of randomised controlled trials
Source: BMC Med. 2005 Oct 17;3:15. doi: 10.1186/1741-7015-3-15 (PMC1274318; doi:10.1186/1741-7015-3-15)
Supplement: Additional File 1 — Search strategy for identification of studies. The Schizophrenia Group's register is based on regular searches of BIOSIS Inside; CENTRAL; CINAHL; EMBASE; MEDLINE and PsycINFO; the hand searching of relevant journals and conference proceedings, and searches of several key grey literature sources. A full description is given in the Group's module on the Cochrane Library. [file 1741-7015-3-15-S1.doc]

1. Electronic searching

1.1. The Cochrane Schizophrenia Group's Register was searched in June 2002 using the phrase:

{[(*anadep* or *chloractil* or *chlorazin* or *chlorpromados* or *chlorpromazine* or *chlorprom-ez-ets* or *(chlor p-z)* or *chromedazine* or *cpz* or *elmarine* or *esmind* or *fenactil* or *hibanil* or *hibernal* or *klorazin* or *klorproman* or *klorpromez* or *largactil* or *megaphen* or *neurazine* or *plegomazine* or *procalm* or *promachel* or *promacid* or *promapar* or *promexin* or *promosol* or *prozil* or *psychozine* or *psylactil* or *serazone* or *sonazine* or *thoradex* or *thorazine* or *tranzine*) in title, abstract, index terms of REFERENCE] or [chlorpromazine* in interventions of STUDY]}

1.2 Details of previous electronic search

Chlorpromazine is known by many names, and the following search phrase was constructed in order to try to aid identification:

(chlorpromazine-phrase) = (anadep or chloractil or chlorazin or chlorpromados or chlorpromazine or chlorprom-ez-ets or (chlor p-z) or chromedazine or cpz or elmarine or esmind or fenactil or hibanil or hibernal or klorazin or klorproman or klorpromez or largactil or megaphen or neurazine or plegomazine or procalm or promachel or promacid or promapar or promexin or promosol or prozil or psychozine or psylactil or (RP near1 4560) or serazone or sonazine or thoradex or thorazine or tranzine).

Relevant randomised trials were identified by searching the following electronic databases:

1.2.1 The Cochrane Schizophrenia Group's Register was searched in October 1999 using the phrase:

(((chlorpromazine-phrase) or (#42 = 5)) and (placebo* or (#42 = 4))).

#42 is the 'Intervention' field and 5 is the code for chlorpromazine; 4 is the code for placebo.

1.2.2 Biological Abstracts (January 1982 to May 1995) was searched using the Cochrane Schizophrenia Group's phrase for both randomised controlled trials and schizophrenia (see: Group search strategy) combined with the phrase:

((chlorpromazine-phrase) and placebo*).

1.2.3 The Cochrane Library (1999, Issue 2) was searched using the phrase:

((chlorpromazine-phrase) and placebo).

1.2.4 EMBASE (January 1980 to May 1995) was searched using the Cochrane Schizophrenia Group's phrase for both randomised controlled trials and schizophrenia (see Group search strategy) combined with the phrase:

(and (chlorpromazine-phrase) and placebo*).

1.2.5 MEDLINE (January 1966 to May 1995) was searched using the Cochrane Schizophrenia Group's phrase for both randomised controlled trials and schizophrenia (see Group search strategy) combined with the phrase:

(and (chlorpromazine-phrase) and placebo*)

1.2.6 PsycLIT (January 1974 to May 1995) was searched using the Cochrane Schizophrenia Group's phrase for both randomised controlled trials and schizophrenia (see Group search strategy) combined with the phrase:

(and (chlorpromazine-phrase) and placebo*)

1.2.7 Local Library Listings of books/university series/dissertations relating to chlorpromazine were searched using the phrase:

(and (chlorpromazine-phrase) and placebo*).

1.2.8 Scisearch (Science Citation Index)

Each of the included studies was sought as a citation on the Scisearch database. Reports of articles that had cited these studies were inspected in order to identify further trials.

2. Reference searching: The references of all identified studies were also inspected for more studies.

3. Personal contact: An attempt was made to contact the first author of each included study for information regarding unpublished trials.

4. Pharmaceutical companies: These were contacted for any unpublished and published trials. Approaches have been made to Rhone Poulenc Rorer, the original developers of chlorpromazine, for access to archive material. Dr R.A Pargiter of Hobart, Tasmania very kindly donated a series of reports from May and Baker (the pharmaceutical company which originally produced chlorpromazine) that listed presentations of work relevant to chlorpromazine and schizophrenia, dating from 1955 to 1973. These were hand searched by BT, CA and JR for further studies.

**Group search strategy**

**Search**

Conference proceedings

American Psychiatric Association
124th Annual Meeting (Boston, USA - May 1968)
131st Annual Meeting (Atlanta, USA - May 1978)
132nd Annual Meeting (Chicago, Illinois May - 1979)
141st Annual Meeting (Quebec, Canada - May 1988)
149th Annual Meeting (USA - May 1996)*
150th Annual Meeting (San Diego, USA - 1997)*
151st Annual Meeting (Toronto, CANADA - 1998)*
152nd Annual Meeting (Washington, USA - 1999)*
153rd Annual Meeting (Illinois, USA - 2000)
154th Annual Meeting (New Orleans 2001)
155th Annual Meeting (2002-Philadelphia)

American Association of Geriatric Psychiatry
11th Annual Meeting (San Diego, CA, USA - March 1998)*

Association of European Psychiatrists
8th Congress (London, UK - July 1996)*
9th Congress (Copenhagen, Denmark - September 1998)*
11th Congress (Stockholm, Sweden - May 2002)

Biological Psychiatry
Sixth World Congress (Nice, France - June 1997)*
Seventh World Congress (Berlin, Germany - 2001)

Collegium Internationale Neuro-psychopharmacologicum
XXth Collegium (Melbourne, Australia - June 1996)*
XXIst Collegium (Glasgow, Scotland, UK - July 1998)*
XXIInd Collegium (Brussels, Belgium - July 2000)*
European College of Neuropsychopharmacology
8th Congress (Venice, Italy - Sept 1995)*
9th Congress (Amsterdam, The Netherlands - Sept 1996)*
10th Congress (Vienna, Austria - Sept 1997)*
11th Congress (Paris, France - 1998)*
12th Congress (London - 1999)*
13th Congress (Munich - 2000)*
14th Congress (Montreal, Canada - 2002)

4th Regional Meeting (Poland - 1999)*
5th Regional Meeting (St Petersburg - 2000)*

European College of Neuropsycopharmacology (ECNP) Congress
Venice (1995)
Amsterdam, The Netherlands (1996)
Vienna (1997)
Paris (1998)
London (1999)
Munich (2000)
Istanbul (2001)

Regional Meetings
Poland (1999)
St Petersburg (2001)

European Federation of Neurological Sciences
4th Congress (Lisbon, Portugal - 1999)

Institute on Psychiatric Services
50th (USA - 1998)
51st (Los Angeles, USA - 1999)
52nd (Philadelphia, USA - 2000)

Royal College of Psychiatrists Winter Meeting
1997 (Cardiff, Wales)
1998 (Belfast)
1999
2000 (Edinburgh)
2001 (London)

Schizophrenia: Breaking down the Barriers
4th International Conference (Vancouver, Canada - October 1996)

Schizophrenia Research
VIth Biennial Winter Workshop (Badgestien, Jan 1992)
VIIth Biennial Winter Workshop (Les Diablerets, Switzerland - Jan 1994)
VIIIth Biennial Winter Workshop (Crans Montana, Switzerland - March 1996)
IXth Biennial Winter Workshop (Davos, Switzerland - Feb 1998)
Xth Biennial Winter Workshop (Davos, Switzerland - Feb 2000)
XIth Biennial Winter Workshop (Davos, Switzerland - Feb - March 2002)

Vth International Congress (Wormsprings, USA - April 1995)
VIth International Congress (Colorado, USA - April 1997)
VIIth International Congress (Santa Fe, New Mexico, USA - April 1999)

World Congress of Psychiatry
Vth Congress (Ciudad de Mexico - December 1971)
Xth Congress (Madrid, Spain - August 1996)*
XIth Congress (Hamburg, Germany - August 1999)

World Congress of Neurology
XVII Congress (London - 2001)

* Conference proceedings are searched using a combination of hand searching and electronic searches. Where proceedings are supplied in electronic form (as marked above with '*') these are searched using the following search phrase:

#1 RANDOMI*
#2 RANDOMLY
#3 (RANDOM* AND ALLOC*)
#4 CROSSOVER
#5 ((SINGL* or DOUBL* or TRIPL* or TREBL*) and (BLIND* or MASK*))
#6 (CLIN* AND TRIAL*)
#7 META-ANALYSIS
#8 (PLACEBO* AND CONTROL*)]
#9 #1 or #2 or #3 or #4 or #5 or #6 or #7 or #8

The results of electronic searches are then hand searched. All reports likely to relate to a trial relevant to the scope of the Group are obtained and added to the Group's Register. All other trials are forwarded to the Cochrane Central Register of Controlled Trials (formerly known as the Cochrane Controlled Trials Register).

**Electronic searches**

a. Biological Abstracts (January 1985 - present)

Trial search phrase:
#1 CLIN*
#2 TRIAL*
#3 #1 near #2
#4 SINGL*
#5 DOUBL*
#6 TREBL*
#7 TRIPL*
#8 BLIND*
#9 MASK*
#10 (#4 or #5 or #6 or #7) near (#8 or #9)
#11 RANDOMI*
#12 RANDOM*
#13 ALLOCAT*
#14 ASSIGN*
#15 #12 near (#13 or #14)
#16 CROSSOVER
#17 #16 or #15 or #11 or #10 or #3

Schizophrenia search phrase:
#1 SCHIZO*
#2 HEBEPHRENI*
#3 OLIGOPHRENI*
#4 PSYCHOTIC*
#5 PSYCHOSIS
#6 PSYCHOSES
#7 CHRONIC*
#8 SEVER*
#9 MENTAL*
#10 ILL*
#11 DISORDER*
#12 ((CHRONIC* or SEVER*) near2 MENTAL*) near2 (ILL* or DISORDER*)
#13 #1 or #2 or #3 or #4 or #5 or #6 or #12
#14 TARDIV*
#15 DYSKINE*
#16 TARDIV* near DYSKINE*
#17 AKATHISI*
#18 ACATHISI*
#19 NEUROLEPTIC*
#20 MALIGNANT
#21 SYNDROME
#22 NEUROLEPTIC* and (MALIGNANT near2 SYNDROME)
#23 MOVEMENT
#24 DISORDER*
#25 #19 and MOVEMENT and DISORDER*
#26 PARKINSONI*
#27 NEUROLEPTIC-INDUC*
#28 PARKINSON'S
#29 DISEASE
#30 PARKINSON'S near1 (DISEASE in TI)
#31 #16 or #17 or #18 or #22 or #25 or #26 or #27
#32 #31 not #30
#33 #32 or #13

The results of electronic searches are then hand searched. All reports likely to relate to a trial relevant to the scope of the Group are obtained and added to the Group's Register. All other trials are forwarded to the Cochrane Central Register of Controlled Trials.

b. Cumulative Index to Nursing and Allied Health Literature (January 1982 - present)

Trial search phrase:
#1 RANDOMI*
#2 CLIN*
#3 TRIAL*
#4 CLIN* near TRIAL*
#5 SINGL*
#6 DOUBL*
#7 TRIPL*
#8 TREBL*
#9 MASK*
#10 BLIND*
#11 (SINGL* or DOUBL* or TRIPL* or TREBL*) and (MASK* or BLIND*)
#12 CROSSOVER
#13 RANDOM*
#14 ALLOCATE*
#15 ASSIGN*
#16 RANDOM* near (ALLOCATE* or ASSIGN*)
#17 "RANDOM-ASSIGNMENT"/ all topical subheadings / all age subheadings
#18 explode "CLINICAL-TRIALS"/ all topical subheadings / all age subheadings
#19 explode "META-ANALYSIS"/ all topical subheadings / all age subheadings
#20 #16 or #12 or #11 or #4 or #1 or #17 or #18 or #19

Schizophrenia search phrase:
#1 explode "PSYCHOSIS"
#2 SCHIZO*
#3 PSYCHOSIS
#4 PSYCHOTIC*
#5 PSYCHOSES
#6 CHRONIC*
#7 SEVER*
#8 MENTAL*
#9 ILL*
#10 DISORDER*
#11 (CHRONIC* or SEVER*) and (MENTAL* and (ILL* or DISORDER*))
#12 OLIGOPHRENI*
#13 HEBEPHRENI*
#14 #1 or #2 or #3 or #4 or #5 or #11 or #12 or #13
#15 TARDIV*
#16 DYSKINE*
#17 TARDIV* near DYSKINE*
#18 AKATHISI*
#19 ACATHISI*
#20 NEUROLEPTIC*
#21 MALIGNANT
#22 SYNDROME
#23 NEUROLEPTIC* and (MALIGNANT near2 SYNDROME)
#24 MOVEMENT
#25 DISORDER*
#26 #20 and MOVEMENT and DISORDER*
#27 PARKINSONI*
#28 NEUROLEPTIC-INDUC*
#29 PARKINSON'S
#30 DISEASE
#31 PARKINSON'S near1 (DISEASE in TI)
#32 #17 or #18 or #19 or #23 or #26 or #27 or #28
#33 #32 not #31
#34 explode "MOVEMENT-DISORDERS"/ all topical subheadings / all age subheadings
#35 "AKATHISIA,-DRUG-INDUCED"/ all topical subheadings / all age subheadings
#36 "DYSKINESIA-DRUG-INDUCED"/ all topical subheadings / all age subheadings
#37 "NEUROLEPTIC-MALIGNANT-SYNDROME"/ all topical subheadings / all age subheadings
#38 #37 or #36 or #35 or #34 or #33
#39 #38 or #14

The results of electronic searches are then hand searched. All reports likely to relate to a trial relevant to the scope of the Group are obtained and added to the Group's Register. All other trials are forwarded to the Cochrane Central Register of Controlled Trials.

c. Cochrane Library (current Issue)

Schizophrenia search phrase:
#1 explode SCHIZOPHRENIA* ME
#2 explode PARANOID-DISORDERS* ME
#3 SCHIZO*
#4 HEBEPHRENI*
#5 OLIGOPHRENI*
#6 PSYCHOTIC*
#7 PSYCHOSIS
#8 PSYCHOSES
#9 CHRONIC*
#10 SEVER*
#11 MENTAL*
#12 ILL*
#13 DISORDER*
#14 ((CHRONIC* or SEVER*) and MENTAL*) and (ILL* or DISORDER*)
#15 #1 or #2 or #3 or #4 or #5 or #6 or #7 or #8 or #14
#16 TARDIV*
#17 DYSKINE*
#18 TARDIV* and DYSKINE*
#19 AKATHISI*
#20 ACATHISI*
#21 NEUROLEPTIC*
#22 MALIGNANT
#23 SYNDROME
#24 NEUROLEPTIC* and (MALIGNANT and SYNDROME)
#25 MOVEMENT
#26 DISORDER*
#27 #21 and MOVEMENT and DISORDER*
#28 PARKINSONI*
#29 NEUROLEPTIC-INDUC*
#30 PARKINSON'S
#31 DISEASE
#32 PARKINSON'S and (DISEASE in TI)
#33 #18 or #19 or #20 or #24 or #27 or #28 or #29
#34 #33 not #32
#35 DYSKINESIA-DRUG-INDUCED* ME
#36 AKATHISIA-DRUG-INDUCED* ME
#37 NEUROLEPTIC-MALIGNANT-SYNDROME* ME
#38 #34 or #35 or #36 or #37
#39 #38 or #15
#40 SCHIZOPHRENIA-AND-DISORDERS-WITH-PSYCHITIC-F* ME
#41 #40 or #39

The results of electronic searches are then hand searched. All reports likely to relate to a trial relevant to the scope of the Group are obtained and added to the Group's Register. All other trials are forwarded to the Cochrane Central Register of Controlled Trials.

d. EMBASE (January 1980 - present)

Trial search phrase:
#1 CLIN*
#2 TRIAL*
#3 #1 near #2
#4 SINGL*
#5 DOUBL*
# 6 TREBL*
# 7 TRIPL*
# 8 BLIND*
# 9 MASK*
# 10 (#4 or #5 or #6 or #7) near (#8 or #9)
# 11 RANDOMI*
# 12 RANDOM*
# 13 ALLOCAT*
# 14 ASSIGN*
#15 #12 near (#13 or #14)
#16 CROSSOVER
#17 #16 or #15 or #11 or #10 or #3
#18 explode "RANDOMIZED-CONTROLLED-TRIAL"/ all subheadings
#19 explode "DOUBLE-BLIND-PROCEDURE"/ all subheadings
#20 explode "CROSSOVER-PROCEDURE"/ all subheadings
# 21 explode "SINGLE-BLIND-PROCEDURE"/ all subheadings
# 22 explode "RANDOMIZATION"/ all subheadings
# 23 #18 or #19 or #20 or #21 or #22 or #17

Schizophrenia search phrase:
#1 SCHIZO*
#2 PSYCHOTIC*
#3 PSYCHOSIS
#4 PSYCHOSES
#5 SCHIZO* or PSYCHOTIC* or PSYCHOSIS or PSYCHOSES
#6 explode "SCHIZOPHRENIA"/ all subheadings
#7 explode "PSYCHOSIS"/ all subheadings
#8 CHRONIC*
#9 SEVERE*
#10 PERSISTENT*
#11 MENTAL*
#12 PSYCHOLOGICAL*
#13 DISORDER*
#14 ILL*
#15 (CHRONIC* or SEVERE* or PERSISTENT*) near (MENTAL* or PSYCHOLOGICAL*) near (DISORDER* or ILL*)
#16 "MENTAL-PATIENT"/ all subheadings
#17 TARDIV*
#18 DYSKINE*
#19 TARDIV* near DYSKINE*
#20 AKATHISI*
#21 NEUROLEPTIC*
#22 MALIGNANT
#23 SYNDROME
#24 NEUROLEPTIC* and (MALIGNANT near2 SYNDROME)
#25 "TARDIVE-DYSKINESIA"/ all subheadings
#26 "AKATHISIA"/ all subheadings
#27 ACATHISIA
#28 "NEUROLEPTIC-MALIGNANT-SYNDROME"/ all subheadings
#29 MOVEMENT
#30 DISORDER*
#31 #21 and MOVEMENT and DISORDER*
#32 #5 or #6 or #7 or #15 or #16
#33 PARKINSONI*
#34 NEUROLEPTIC-INDUCED
#35 #19 or #20 or #24 or #25 or #26 or #27 or #28 or #31 or #33 or #34
#36 PARKINSON'S
#37 #35 not (PARKINSON'S in TI)
#38 #37 or #32

The results of electronic searches are then hand searched. All reports likely to relate to a trial relevant to the scope of the Group are obtained and added to the Group's Register. All other trials are forwarded to the Cochrane Central Register of Controlled Trials.

e. LILACS (January 1982 - August 1996)

Search phrase:
#1 RANDOM$
#2 ALEATORI$ or CASUAL or ACASO or AZAR
#3 ((DUPLO or DOBLE or SIMPLE or TRIPLO or TRIPLE) and (CEGO or CIEGO))
#4 ((DOUBL$ or SINGL$ or TRIPL$ or TREBL$) and (BLIND$ or MASK$)
#5 SINGLE-MASKED STUDY/
#6 DOUBLE-MASKED STUDY/
#7 PROPHYLATIC CONTROLLED TRIALS/
#8 PLACEBO$ and CONTROL$
#9 CLINICAL$ and TRIAL$
#10 #1 or #2 or #3 or #4 or #5 or #6 or #7 or #8 or #9

The results of electronic searches are then hand searched. All reports likely to relate to a trial relevant to the scope of the Group are obtained and added to the Group's Register. All other trials are forwarded to the Cochrane Central Register of Controlled Trials.

f. MEDLINE (January 1966 - present)

Trial search phrase:
#1 RANDOMIZED
#2 CONTROLLED
#3 TRIAL
#4 RANDOMIZED CONTROLLED TRIAL in PT
#5 RANDOMIZED-CONTROLLED-TRIALS / ALL
#6 RANDOM-ALLOCATION / ALL
#7 DOUBLE-BLIND-METHOD / ALL
#8 SINGLE-BLIND-METHOD / ALL
#9 CLINICAL
#10 TRIAL
#11 CLINICAL TRIAL in PT
#12 explode CLINICAL-TRIALS / ALL
#13 CLIN*
#14 TRIAL*
#15 #13 near #14
#16 SINGL*
#17 DOUBL*
#18 TREBL*
#19 TRIPL*
#20 BLIND*
#21 MASK*
#22 (#16 or #17 or #18 or #19) near (#20 or #21)
#23 RANDOM*
#24 ALLOCATE*
#25 CONTROLLED CLINICAL TRIAL IN PT
#26 RANDOMI*
#27 ASSIGN*
#28 #23 near (#24 or #27)
#29 CROSSOVER
#30 #29 or #28 or #26 or #25 or #22 or #15 or #12 or #11 or #8 or #7 or #6 or #5 or #4

Schizophrenia search phrase:
#1 explode "SCHIZOPHRENIA" / all subheadings
#2 explode "PARANOID-DISORDERS" / all subheadings
#3 SCHIZO*
#4 HEBEPHRENI*
#5 OLIGOPHRENI*
#6 PSYCHOTIC*
#7 PSYCHOSIS
#8 PSYCHOSES
#9 CHRONIC*
#10 SEVER*
#11 MENTAL*
#12 ILL*
#13 DISORDER*
#14 ((CHRONIC* or SEVER*) near2 MENTAL*) near2 (ILL* or DISORDER*)
#15 #1 or #2 or #3 or #4 or #5 or #6 or #7 or #8 or #14
#16 TARDIV*
#17 DYSKINE*
#18 TARDIV* near DYSKINE*
#19 AKATHISI*
#20 ACATHISI*
#21 NEUROLEPTIC*
#22 MALIGNANT
#23 SYNDROME
#24 NEUROLEPTIC* and (MALIGNANT near2 SYNDROME)
#25 MOVEMENT
#26 DISORDER*
#27 #21 and MOVEMENT and DISORDER*
#28 PARKINSONI*
#29 NEUROLEPTIC-INDUC*
#30 PARKINSON'S
#31 DISEASE
#32 PARKINSON'S near1 (DISEASE in TI)
#33 #18 or #19 or #20 or #24 or #27 or #28 or #29
#34 #33 not #32
#35 "DYSKINESIA-DRUG-INDUCED"/ all subheadings
#36 "AKATHISIA-DRUG-INDUCED"/ all subheadings
#37 "NEUROLEPTIC-MALIGNANT-SYNDROME"/ all subheadings
#38 #34 or #35 or #36 or #37
#39 #38 or #15

The results of electronic searches are then hand searched. All reports likely to relate to a trial relevant to the scope of the Group are obtained and added to the Group's Register. All other trials are forwarded to the Cochrane Central Register of Controlled Trials.

g. PsycINFO (January 1876 - present) [Previously PsycLIT]

Trial search phrase:
#1 RANDOMI*
#2 SINGL*
#3 DOUBL*
#4 TREBL*
#5 TRIPL*
#6 BLIND*
#7 MASK*
#8 (#2 or #3 or #4 or #5) near (#6 or #7)
#9 CLIN*
#10 TRIAL*
#11 #9 near #10
#12 PLACEBO*
#13 PLACEBO- in DE
#14 CROSSOVER
#15 TREATMENT-EFFECTIVENESS-EVALUATION in DE
#16 MENTAL-HEALTH-PROGRAM-EVALUATION in DE
#17 RANDOM*
#18 ASSIGN*
#19 ALLOCATE*
#20 #17 near (#18 or #19)
#21 #20 or #16 or #15 or #14 or #13 or #12 or #11 or #8 or #1

Schizophrenia search phrase:
#1 SCHIZO*
#2 HEBEPHRENI*
#3 OLIGOPHRENI*
#4 PSYCHOTIC*
#5 PSYCHOSIS
#6 PSYCHOSES
#7 CHRONIC*
#8 SEVER*
#9 MENTAL*
#10 ILL*
#11 DISORDER*
#12 ((CHRONIC* or SEVER*) near2 MENTAL*) near2 (ILL* or DISORDER*)
#13 explode "SCHIZOPHRENIA"
#14 explode "PSYCHOSIS"
#15 explode "SCHIZOAFFECTIVE-DISORDER"
#16 #1 or #2 or #3 or #4 or #5 or #6 or #12 or #13 or #14 or #15
#17 TARDIV*
#18 DYSKINE*
#19 TARDIV* near DYSKINE*
#20 AKATHISI*
#21 ACATHISI*
#22 NEUROLEPTIC*
#23 MALIGNANT
#24 SYNDROME
#25 NEUROLEPTIC* and (MALIGNANT near2 SYNDROME)
#26 MOVEMENT
#27 DISORDER*
#28 #22 and MOVEMENT and DISORDER*
#29 PARKINSONI*
#30 NEUROLEPTIC-INDUC*
#31 PARKINSON'S
#32 DISEASE
#33 PARKINSON'S near1 (DISEASE in TI)
#34 #19 or #20 or #21 or #25 or #28 or #29 or #30
#35 #34 not #33
#36 "NEUROLEPTIC-MALIGNANT-SYNDROME" IN DE
#37 explode "DYSKINESIA"
#38 explode "AKATHISIA"
#39 "PARKINSONISM-" IN DE
#40 #35 or #36 or #37 or #38 or #39
#41 #40 or #16

The results of electronic searches are then hand searched. All reports likely to relate to a trial relevant to the scope of the Group are obtained and added to the Group's Register. All other trials are forwarded to the Cochrane Central Register of Controlled Trials.

h. PSYNDEX (January 1977 - September 2002)

Search phrase:
#1 RANDOM* and (ALLOCAT* or ASSIGN*)
#2 RANDOMI*
#3 (DOUBL* or SINGL* or TRIPL* or TREBL*) near (BLIND* or MASK*)
#4 DOPPELBLIND*
#5 PLA?EBO* and ((EITHER or ENTWEDER) or (TREAT* or BEHAND* or UNTERSUCH*))
#6 PLA?EBO* near ((VS or VERSUS) or VERUM)
#7 [ZUFA?LL* and (EXPERIMENT* or EVALU* or EFFE?T*) and TREAT*]
#8 ZUGEWIESEN and KONTROLLGRUPPE*
#9 #1 or #2 or #3 or #4 or #5 or #6 or #7 or #8

The results of electronic searches are then hand searched. All reports likely to relate to a trial relevant to the scope of the Group are obtained and added to the Group's Register. All other trials are forwarded to the Cochrane Central Register of Controlled Trials.

i. System for Information on the Grey Literature of Europe (January 1980 - present)

Trial search phrase:
#1 CLIN*
#2 TRIAL*
#3 #1 near #2
#4 SINGL*
#5 DOUBL*
#6 TREBL*
#7 TRIPL*
#8 BLIND*
#9 MASK*
#10 (#4 or #5 or #6 or #7) near (#8 or #9)
#11 RANDOMI*
#12 RANDOM*
#13 ALLOCAT*
#14 ASSIGN*
#15 #12 near (#13 or #14)
#16 CROSSOVER
#17 #16 or #15 or #11 or #10 or #3

Schizophrenia search phrase:
#1 SCHIZO*
#2 HEBEPHRENI*
#3 OLIGOPHRENI*
#4 PSYCHOTIC*
#5 PSYCHOSIS
#6 PSYCHOSES
#7 CHRONIC*
#8 SEVER*
#9 MENTAL*
#10 ILL*
#11 DISORDER*
#12 ((CHRONIC* or SEVER*) near2 MENTAL*) near2 (ILL* or DISORDER*)
#13 #1 or #2 or #3 or #4 or #5 or #6 or #12
#14 TARDIV*
#15 DYSKINE*
#16 TARDIV* near DYSKINE*
#17 AKATHISI*
#18 ACATHISI*
#19 NEUROLEPTIC*
#20 MALIGNANT
#21 SYNDROME
#22 NEUROLEPTIC* and (MALIGNANT near2 SYNDROME)
#23 MOVEMENT
#24 DISORDER*
#25 #19 and MOVEMENT and DISORDER*
#26 PARKINSONI*
#27 NEUROLEPTIC-INDUC*
#28 PARKINSON'S
#29 DISEASE
#30 PARKINSON'S near1 (DISEASE in TI)
#31 #16 or #17 or #18 or #22 or #25 or #26 or #27
#32 #31 not #30
#33 #32 or #13

The results of electronic searches are then hand searched. All reports likely to relate to a trial relevant to the scope of the Group are obtained and added to the Group's Register. All other trials are forwarded to the Cochrane Central Register of Controlled Trials.

j. Sociofile (January 1973 - present)

Trial search phrase:
#1 RANDOMI*
#2 CLIN*
#3 TRIAL*
#4 CLIN* near TRIAL*
#5 SINGL*
#6 DOUBL*
#7 TRIPL*
#8 TREBL*
#9 MASK*
#10 BLIND*
#11 (SINGL* or DOUBL* or TRIPL* or TREBL*) and (MASK* or BLIND*)
#12 CROSSOVER
#13 RANDOM*
#14 ALLOCATE*
#15 ASSIGN*
#16 RANDOM* near (ALLOCATE* or ASSIGN*)
#17 #16 or #12 or #11 or #4 or #1
Schizophrenia search phrase:
#1 explode "SCHIZOPHRENIA"
#2 explode "PSYCHOSIS"
#3 SCHIZO*
#4 PSYCHOSIS
#5 PSYCHOTIC*
#6 PSYCHOSES
#7 CHRONIC*
#8 SEVER*
#9 MENTAL*
#10 ILL*
#11 DISORDER*
#12 (CHRONIC* or SEVER*) and (MENTAL* and (ILL* or DISORDER*))
#13 OLIGOPHRENI*
#14 HEBEPHRENI*
#15 #1 or #2 or #3 or #4 or #5 or #6 or #12 or #13 or #14
#16 TARDIV*
#17 DYSKINE*
#18 TARDIV* near DYSKINE*
#19 AKATHISI*
#20 ACATHISI*
#21 NEUROLEPTIC*
#22 MALIGNANT
#23 SYNDROME
#24 NEUROLEPTIC* and (MALIGNANT near2 SYNDROME)
#25 MOVEMENT
#26 DISORDER*
#27 #21 and MOVEMENT and DISORDER*
#28 PARKINSONI*
#29 NEUROLEPTIC-INDUC*
#30 PARKINSON'S
#31 DISEASE
#32 PARKINSON'S near1 (DISEASE in TI)
#33 #18 or #19 or #20 or #24 or #27 or #28 or #29
#34 #33 not #32
#35 #34 or #15

The results of electronic searches are then hand searched. All reports likely to relate to a trial relevant to the scope of the Group are obtained and added to the Group's Register. All other trials are forwarded to the Cochrane Central Register of Controlled Trials.

**Hand searching**

The following journals have been/are being searched and relevant trials are entered into the Register:

Acta Psychiatrica Scandinavica (1948 - ongoing)
Acta Psychiatrica Scandinavica Supplementum (1948 - ongoing)
American Journal of Psychiatry (1948 - ongoing)
Arab Journal of Psychiatry (1990-97)
Archives of General Psychiatry ( 1959 - ongoing)
Australian and New Zealand Journal of Psychiatry (1967 - ongoing)
Behaviour Therapy (1971, 76, 81, 86, 91)
Biological Psychiatry (1994-1995)
British Journal of Psychiatry (1963 - ongoing)
Canadian Journal of Psychiatry (1979 - ongoing)
Clinical Pharmacology and Therapeutics (1971, 76, 81, 86, 91)
Canadian Psychiatric Association Journal (continued as Canadian Journal of Psychiatry (1956-1978)
Der Nervenarzt (1980-95)
Egyptian Journal of Mental Health (1988, 90-92)
Egyptian Journal of Psychiatry (1978-83, 86, 88-89)
Hospital and Community Psychiatry (continued as Psychiatric Services) (1966-1994)
Journal of Consulting and Clinical Psychology (1980 - 1995)
Journal of Intellectual Disability Research (1992 - 2000)
Journal of Mental Deficiency Research (continued as Journal of Intellectual Disability Research) (1958-1991)
Journal of Mental Science (continued as British Journal of Psychiatry) (1948-1962)
Journal of Nervous and Mental Disease (1948-1993)
Pakistan Journal of Clinical Psychiatry (1991- ongoing)
Polish Journal of Psychiatry (1980-1994)
Psychiatria Fennica (1970- ongoing)
Psychiatric Services (1995 - ongoing)
Psychological Medicine (1970 - 1997)
Psychosomatic Medicine (1971, 76, 81, 86, 91)
Schizophrenia Bulletin (1969 - ongoing)
Schizophrenia Research (1988 - ongoing)
Zhurnal Nevropatologii I Psikhiatrii Imeni SS Korsakov (1980-1995)
